# Supplementary material for: Spatial coherence of room-temperature monolayer WSe2 exciton-polaritons in a trap
Source: Nat Commun. 2021 Nov 4;12:6406. doi: 10.1038/s41467-021-26715-9 (PMC8569157; doi:10.1038/s41467-021-26715-9)
Supplement: Supplementary file 1 — Supplementary Information [file 41467_2021_26715_MOESM1_ESM.pdf]

Supplementary information: Spatial coherence of room-temperature monolayer WSe_2 exciton-polaritons in a trap

Hangyong Shan[1,†], Lukas Lackner[1], Bo Han[1], Evgeny Sedov[2,3,4], Christoph Rupprecht[5], Heiko Knopf[6,7,8], Falk Eilenberger[6,7,8], Johannes Beierlein[5], Nils Kunte[1], Martin Esmann[1], Kentaro Yumigeta[9], Kenji Watanabe[10], Takashi Taniguchi[11], Sebastian Klembt[5], Sven Höfling[5], Alexey V. Kavokin[2,3,12], Sefaattin Tongay[9,†], Christian Schneider[1,†] and Carlos Anton-Solanas[1,†]

[1]Institute of Physics, Carl von Ossietzky University, 26129 Oldenburg, Germany.
[2]School of Science, Westlake University, 18 Shilongshan Road, Hangzhou 310024, Zhejiang Province, People's Republic of China
[3]Institute of Natural Sciences, Westlake Institute for Advanced Study, 18 Shilongshan Road, Hangzhou 310024, Zhejiang Province, People's Republic of China
[4]Vladimir State University named after A. G. and N. G. Stoletovs, Gorky str. 87, 600000, Vladimir, Russia
[5]Technische Physik, Universität Würzburg, D-97074 Würzburg, Am Hubland, Germany.
[6]Institute of Applied Physics, Abbe Center of Photonics, Friedrich Schiller University, 07745 Jena, Germany.
[7]Fraunhofer-Institute for Applied Optics and Precision Engineering IOF, 07745 Jena, Germany.
[8]Max Planck School of Photonics, 07745 Jena, Germany.
[9]School for Engineering of Matter, Transport, and Energy, Arizona State University, Tempe, Arizona 85287, USA
[10]Research Center for Functional Materials, National Institute for Materials Science, 1-1 Namiki, Tsukuba 305-0044, Japan
[11]International Center for Materials Nanoarchitectonics, National Institute for Materials Science, 1-1 Namiki, Tsukuba 305-0044, Japan
[12]Russian Quantum Center, Skolkovo IC, Bolshoy Boulevard 30, bld. 1, 121205, Moscow, Russia
[†]Corresponding author. Email: hangyong.shan@uni-oldenburg.de, sefaattin.tongay@asu.edu, christian.schneider@uni-oldenburg.de, carlos.anton-solanas@uni-oldenburg.de

S1. The coupled oscillator model and simulation of the polariton dispersion relation

We describe the upper and lower polariton dispersion relation using the simple two-coupled oscillator model. In this frame, the eigen-energies of the upper and lower polariton dispersion relations are:

$$E_{UP,LP}(k_{||}) = \frac{1}{2}\left(E_{ex} + E_{cav} \pm \sqrt{V^2 + (E_{cav} - E_{exc})^2}\right) \quad (1.1)$$

where E_x and E_c (E_{UP} and E_{LP}) represent the exciton and photonic cavity (lower and upper polariton) energies, respectively, and V is the normal mode splitting.

To support the measured dispersion relation described in Fig. 1c, we calculate the energy spectrum of polaritons in the structure. The continuous part of the spectrum for a large wave vector magnitude close to or exceeding 2 μm^{-1} corresponds to the dispersion of quasi-free 2D polaritons. To take into account coexistence of the continuous spectrum and the discrete spectrum of trapped particles[1], we solve the Schrödinger equation $[\hat{T} + V(x)]\psi(x) = E\psi(x)$ for a polariton wave function $\psi(x)$ in a confining potential $V(x)$ of a finite width and depth[2].

The size of the potential trap is 10 x 7 μm^2. In our calculations, we take into account the asymmetry of the confining potential due to the complex shape of the monolayer flake. The best fit is achieved for the depth of the potential of 15 meV. $\hat{T}$ is the kinetic energy operator describing dispersion of free polaritons. The calculation of the discrete part of the spectrum is completed by taking into account the PL intensity and broadening of the eigenmodes.

Taking homogeneous broadening of a photonic fraction of each mode *j* with the Lorentz profile $f_j \propto \left[(E - E_j)^2 + \gamma_{\rm ph}^2\right]^{-1}$ of width $\gamma_{\rm ph}$, we find the intensity of the mode as follows[3]: $I_j \propto |$

$C_j|^2 f_j \exp(-E_j/k_B T)$, where C_j is the photonic Hopfield coefficient. The resulting simulated spectrum is presented in Fig. 1d.

S2. Modelling the input-output curve of room temperature TMD polaritons

For simulating polariton relaxation, we use the Boltzmann equation[4]:

$$d_t n_{\mathbf{k}} = P_{\mathbf{k}} - \Gamma_{\mathbf{k}} n_{\mathbf{k}} - n_{\mathbf{k}} \sum_{\mathbf{k}'} W_{\mathbf{k}\to\mathbf{k}'}(1+n_{\mathbf{k}'}) + (1+n_{\mathbf{k}}) \sum_{\mathbf{k}'} W_{\mathbf{k}'\to\mathbf{k}} n_{\mathbf{k}'}, \tag{2.1}$$

where $n_{\mathbf{k}}$ is the occupation number of a $\mathbf{k}$ state, $P_{\mathbf{k}}$ and $\Gamma_{\mathbf{k}}$ are the pump and the decay rate of the $\mathbf{k}$ state. The polariton decay rate is found as $\Gamma_{\mathbf{k}} = |C_{\mathbf{k}}|^2\gamma_C + |X_{\mathbf{k}}|^2\gamma_X$, where γ_C and γ_X are the cavity photon and exciton decay rates, $C_{\mathbf{k}}$ and $X_{\mathbf{k}}$ are the Hopfield coefficients that determine the photon and exciton fractions:

$$X_{\mathbf{k}} = \frac{1}{2}\left(1 + \delta_{\mathbf{k}}\Big/\sqrt{\delta_{\mathbf{k}}^2 + V_R^2}\right)^{1/2}, \qquad C_{\mathbf{k}} = \frac{1}{2}\left(1 - \delta_{\mathbf{k}}\Big/\sqrt{\delta_{\mathbf{k}}^2 + V_R^2}\right)^{1/2}, \tag{2.2}$$

where $\delta_{\mathbf{k}} = (E_{C0} - E_X) + \hbar^2 k^2/2m_C$ is the exciton-photon detuning, E_X is the exciton energy, E_{C0} is the bottom of the dispersion of cavity photons, m_C is the effective mass of cavity photons, V_R is the Rabi splitting energy. In Eq. (2.1), $W_{\mathbf{k}\to\mathbf{k}'}$ is the scattering rate from the $\mathbf{k}$ state to the $\mathbf{k}'$ state. We consider two mechanisms of scattering $W_{\mathbf{k}\to\mathbf{k}'} = W_{\mathbf{k}\to\mathbf{k}'}^{\mathrm{phon}} + W_{\mathbf{k}\to\mathbf{k}'}^{\mathrm{pp}}$: the phonon-mediated scattering $W_{\mathbf{k}\to\mathbf{k}'}^{\mathrm{phon}}$ and the polariton-polariton scattering $W_{\mathbf{k}\to\mathbf{k}'}^{\mathrm{pp}}$, which are found as follows:

$$W_{\mathbf{k}\to\mathbf{k}'}^{\mathrm{phon}} = \frac{|G(\mathbf{k}-\mathbf{k}')|^2}{2\pi\rho u S} |X_{\mathbf{k}}|^2 |X_{\mathbf{k}'}|^2 \int \sqrt{|\mathbf{k}-\mathbf{k}'|^2 + q_z^2} \left[\left(\frac{1}{2} \mp \frac{1}{2}\right) + N_{\mathbf{k}-\mathbf{k}',q_z}^{\mathrm{phon}}\right]$$
$$\times \frac{\hbar\gamma_{\mathbf{k}'}}{\left[E(\mathbf{k}') - E(\mathbf{k}) \mp E^{\mathrm{phon}}(\mathbf{k}-\mathbf{k}', q_z)\right]^2 + (\hbar\gamma_{\mathbf{k}'})^2} dq_z, \tag{2.3a}$$

$$W_{\mathbf{k}\to\mathbf{k}'}^{\mathrm{pp}} = \frac{S}{2\pi\hbar} \int d\mathbf{q} |M_X|^2 X_{\mathbf{k}} X_{\mathbf{k}'} X_{\mathbf{q}} X_{\mathbf{q}+\mathbf{k}'-\mathbf{k}} N_{\mathbf{q}} \left[1 + N_{\mathbf{q}+\mathbf{k}'-\mathbf{k}}\right]$$
$$\frac{\hbar\gamma_{\mathbf{k}'}\pi^{-1}}{[E(\mathbf{k}') - E(\mathbf{k}) + E(\mathbf{q}+\mathbf{k}'-\mathbf{k}) - E(\mathbf{q})]^2 + (\hbar\gamma_{\mathbf{k}'})^2}, \tag{2.3b}$$

where

$$G(\mathbf{k}-\mathbf{k}') \simeq D_e I_e^{\parallel}(\mathbf{k}-\mathbf{k}') - D_h I_h^{\parallel}(\mathbf{k}-\mathbf{k}') \tag{2.4}$$

with the exciton-phonon overlap integrals

$$I_{e,h}^{\parallel}(\mathbf{k}-\mathbf{k}') = \left[1 + \left(\frac{m_{h,e}|\mathbf{k}-\mathbf{k}'| a_B^{2D}}{2(m_e + m_h)}\right)^2\right]^{-3/2}. \tag{2.5}$$

$D_{e,h}$ are the deformation coefficients of the conduction band and valence band, $m_{e,h}$ are the electron and hole effective masses, a_B^{2D} is the exciton Bohr radius in the monolayer. In Eq. (2.3), ρ is the density of the material, u is the speed of the longitudinal acoustic mode, S is the quantization area. $\mathbf{k}$ is the in-plane wave vector, q_z is the normal-to-plane wave number. $N_{\mathbf{q}}^{\mathrm{phot}}$ is the equilibrium distribution of phonons, $E^{\mathrm{phon}}(\mathbf{k}-\mathbf{k}', q_z)$ is the phonon dispersion, $\hbar\gamma_{\mathbf{k}}$ is the energy width of the polariton $\mathbf{k}$ state. In Eq. (2.3a), "−" is for absorption ($E(\mathbf{k}) < E(\mathbf{k}')$), while "+" is for emission of a phonon ($E(\mathbf{k}) > E(\mathbf{k}')$). The cut off value of q_z in the integral in Eq. (2.3a) is $2\pi/L_z$, where L_z is the average displacement of electrons and holes in the direction perpendicular to the monolayer. It can be estimated as the thickness of the monolayer. The exciton-exciton interaction constant is $M_X \approx 6\left(a_B^{2D}\right)^2 E_b/S$, E_b is the exciton binding energy. To get closer to the experimental conditions when calculating the dependence of the polariton states on the pump power, we take into account the polariton ensemble fluctuations by averaging of the scattering parameters amplified by stimulated processes.

For simulations we use the following values of the parameters taken mostly from[5]: exciton energy is $E_X = 1.67\ \mathrm{eV}$, bottom of the cavity photon dispersion is $E_{C0} = 1.612\ \mathrm{eV}$, effective mass of cavity photons is $m_C = 0.85 \times 10^{-5} m_e$, Rabi splitting is $V_R = 30\ \mathrm{meV}$. Cavity photon and exciton decay rates are $\gamma_C = 0.6\ \mathrm{ps}^{-1}$, $\gamma_X = 1\ \mathrm{ps}^{-1}$, electron and hole masses are $m_e = 0.64 m_0$, $m_h = 0.71 m_0$, speed of sound is $u = 4.1 \times 10^5\ \mathrm{cm\ s}^{-1}$; density of the solid is $\rho = 6 \times 10^3\ \mathrm{kg\ m}^{-3}$, the deformation coefficients are $D_e = 3.4\ \mathrm{eV}$, $D_h = 2.8\ \mathrm{eV}$, the width of the Lorentzian is $\hbar\gamma_{\mathbf{k}} = \hbar\Gamma_k/2$, the 2D Bohr radius is $a_B^{2D} = 0.8\ \mathrm{nm}$, the binding energy is $E_b = 0.8\ \mathrm{eV}$. Temperature is taken as $T = 300\ \mathrm{K}$. The spatial scales are $L_z = 0.335\ \mathrm{nm}$ and $S = 70\ \mu\mathrm{m}^2$.

S3. Additional data on the input-output curve

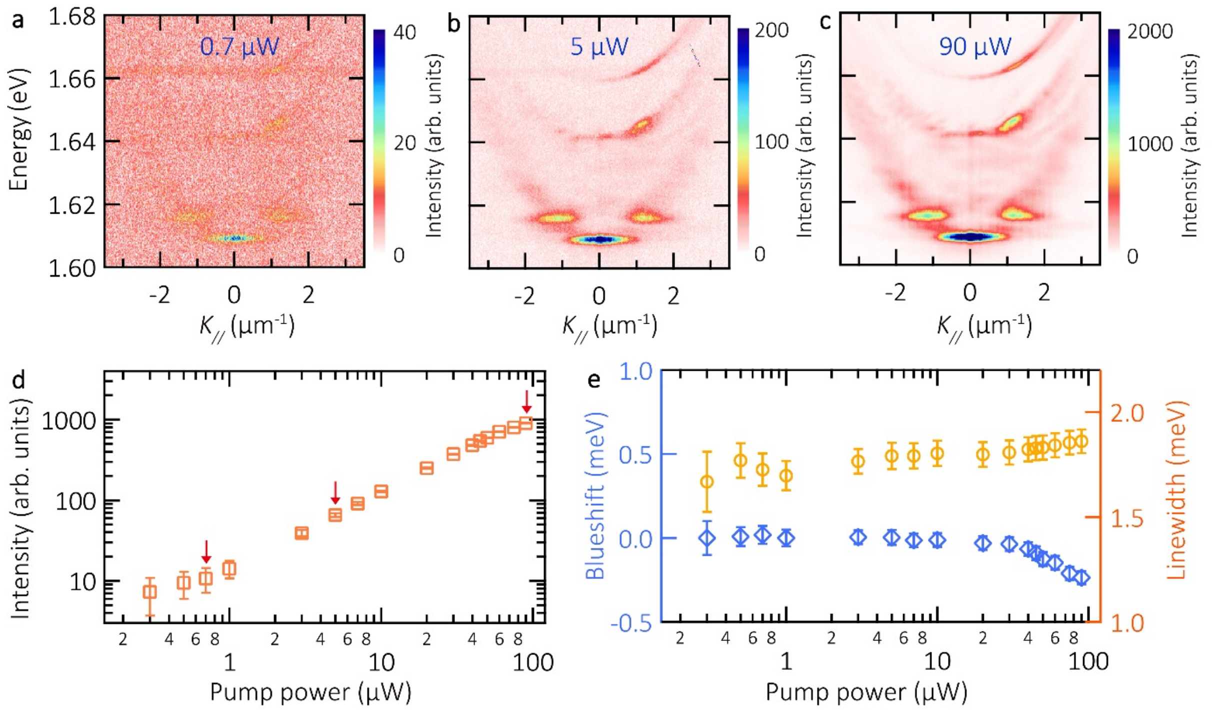

Supplementary Figure 1. Density-dependent properties of room temperature exciton-polaritons with a recording sequence of power decrement. a-c Polariton dispersion relations under 0.7, 5 and 90 μW pump power excitation. Herein, the experiment was first performed under 90 μW, and the pump power was gradually decreased till 0.3 μW. It is in a reverse recording sequence in comparison with the data acquired in Fig. 3. **d** Integrated PL emission intensity as a function of pump power, plotted in a double-logarithmic scale. The arrows indicate the corresponding dispersion relations shown in panels a-c. The error bars are obtained by comparing the polariton signal intensity to the standard deviation of the background noise. **e** Corresponding blueshift (blue diamonds) and linewidth (orange circles) of exciton-polaritons as a function of pump power. The blueshift and linewidth error bars correspond to the 95% confidence interval of the peak fitting.

S4. Additional data on the fine structure splitting of the polariton trap and DOLP of the emission

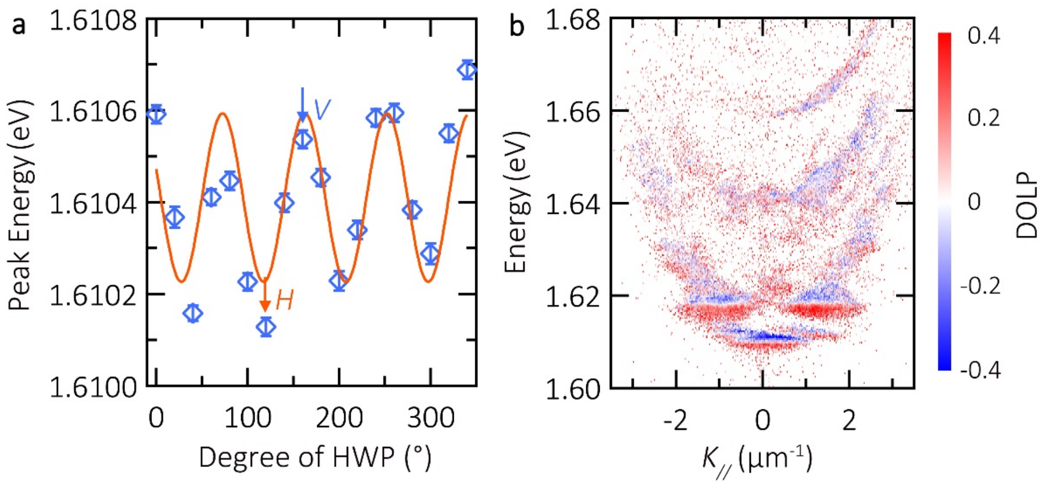

Supplementary Figure 2. Study on the two linearly polarised modes of the polariton trap. a Spectral position of the trap versus the analysis of linear polarisation of the emission. The labels H and V correspond to the analysis of the H and V modes of the trap, where the energy splitting between H and V is maximum. The sinusoidal fit indicates a fine structure splitting of 0.40±0.03 meV. The error bars of peak energy correspond to the 95% confidence interval of the peak fitting. **b** Corresponding DOLP of the dispersion relation, calculated as (I_H-I_V)/(I_H+I_V), considering the dispersion relations H and V, labelled in panel a.

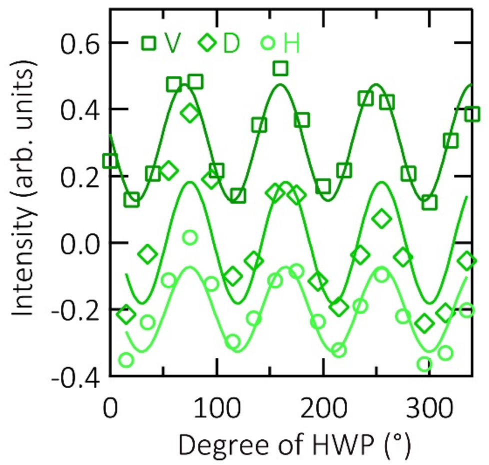

Supplementary Figure 3. Intensity of the polariton trap as a function of the HWP angle, for three different orientations of the input laser polarisation (see V-vertical, D-diagonal and H-horizontal orientations). Similar DOLP is observed in the three cases.

S5. Spectral tomography of the polariton trap in real space

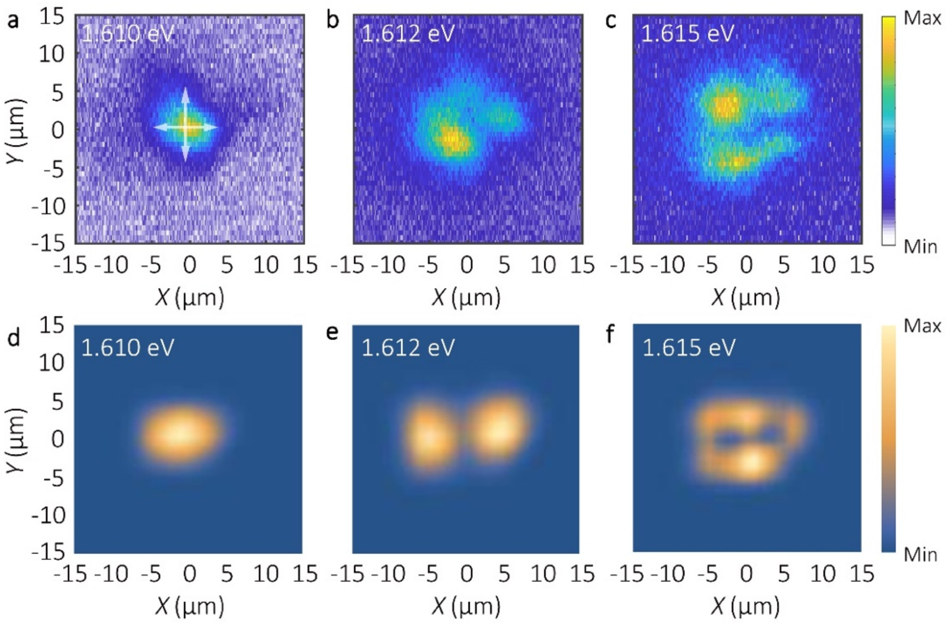

Supplementary Figure 4. Spectral tomography of the polariton trap, showing the full distribution of the emission in real space at different energy values: **a** 1.610, **b** 1.612, and **c** 1.615 eV. In panel **a**, the horizontal and vertical arrows (7 and 10 µm, respectively) indicate the dimensions of the polariton trap. **d**-**f** Corresponding simulation results based on the model of polariton dispersion relation in supplementary material section S1.

In Fig. 4b of the main text, below 1.62 eV, we identify three polariton states at energies: 1.617, ~1.612 and 1.610 eV. However, Figs. 3a-c seem to displays only two energy states at 1.617 and 1.610 eV. We would like to remark that Figs. 3a-c [4b,c] show the polariton emission map versus energy and momentum [real] space. Polaritons may present a different intensity distribution in each of the real and reciprocal spaces when a single slice at *X*=0 [$K_{//}$=0] of the full real [momentum] distribution is spectrally resolved. This is the case here, see Supplementary Fig. 5, where we adapt the false colour scale intensity to highlight the presence of the state at 1.612 eV, which appears much weaker in the single slice of momentum space at $K_{//}$=0. This polariton state at 1.612 eV is also reproduced by the simulated dispersion relation in the Fig. 1d of the main text.

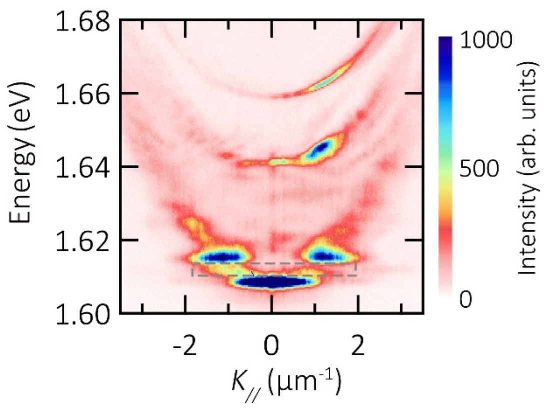

Supplementary Figure 5. Detail on the polariton modal emission. The figure is reproduced from Fig. 3c with an adapted colour scale to highlight the faint polariton mode at ~1.612 eV.

S6. Supplementary data on the coherence measurements: time-delay and power dependence

We notice that the peak of $<G^{(1)}>$ at zero delay in Fig. 4e is smaller than the expected value in Fig. 4f for the corresponding pump power (90 μW). We attribute this mismatch to a slight spatial displacement (few microns) between the two interference arms in the case of the temporal dependence with respect to the pump power dependence experiments.

It is important to note that the $G^{(1)}$ extracted from the contrast of interference fringes characterises the coherence length in plane of the sample. It is different from the coherence length in the propagation direction of the emitted light, which would be simply proportional to the coherence time. The qualitative difference of pump power dependencies of the coherence time and the in-plane coherence length is a characteristic feature of polariton lasers.

The coherence below threshold phenomenon is not surprising in time-integrated measurements, and might be a manifestation of the fluctuation-dominated regime of polariton lasing studied both theoretically and experimentally [2,5]. In this regime, a coherent polariton condensate is characterised by a finite life-time, so that in the case of cw-pumping the system is part-time coherent. The contribution of the coherent fraction to the time-integrated interferometry is small but not negligible below the polariton lasing threshold, and it is large but not 100% above threshold. The threshold itself is not a point but rather a range of pump intensities in this case. Fluctuations of the polariton population are caused by several factors including the fluctuations of the pump intensity, dynamical fluctuations in the processes of exciton formation and exciton energy relaxation to the polariton mode, fluctuation of the condensate depletion rates.

Indeed, in the previous literature of polariton condensation, it is common to observe weak interference fringes below threshold power. For example, as reported in Ref. [6], weak interference is observed when the power is below the threshold ($0.2P_{th}$). Also, in Ref. [7], a weak but visible interference pattern is observed for a pump power of $0.6P_{th}$.

Studies in CdTe microcavities reported multimode-confinement and coherence of trapped polariton states at different energies[8]. The coherence recorded at higher energies in our experiments shows a comparable scenario, see regions highlighted in Supplementary Fig. 10. Importantly, we would like to remark the fact that those high-energy states display a very low interference contrast, smaller than 3%, which decays on timescales <3 ps. In particular, the coherent fraction is very low, compared to the ground-state. We thus believe that this result is not of critical relevance for the main message of the paper and we rather present the coherence studies in the low energy states, opposed to an in-depth discussion and analysis of fluctuations on the <5% level in higher energy modes.

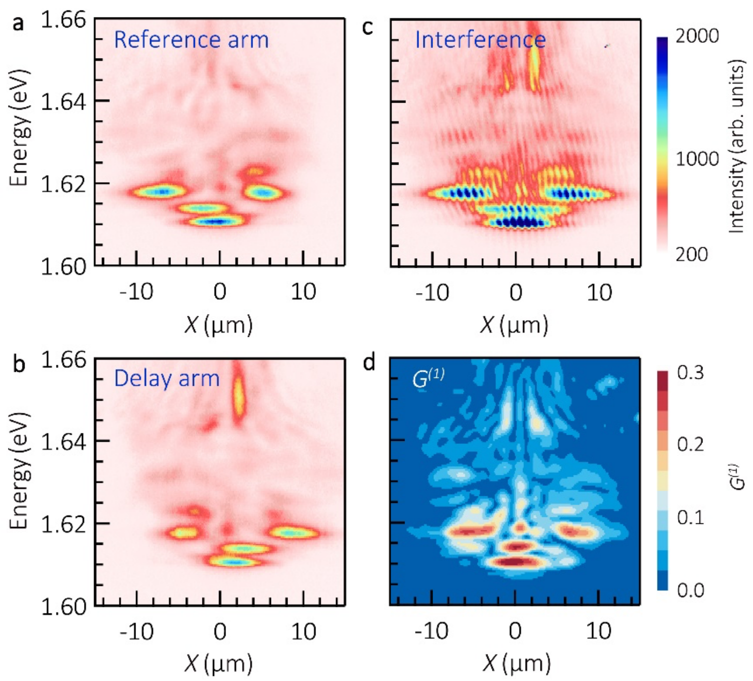

Supplementary Figure 6. First order autocorrelation measurement of room temperature exciton-polaritons. a, b PL distribution of polaritons as a function of energy and real space, recorded from the reference arm and delay arm of Michelson interferometer, respectively. The pump power is 100 μW and the delay time is 0 ps. **c** Corresponding interference image, which is induced by the coherent interaction of two arms (panels a, b) of Michelson interferometer. **d** Corresponding first order correlation function $G^{(1)}$ encoded in a false color scale.

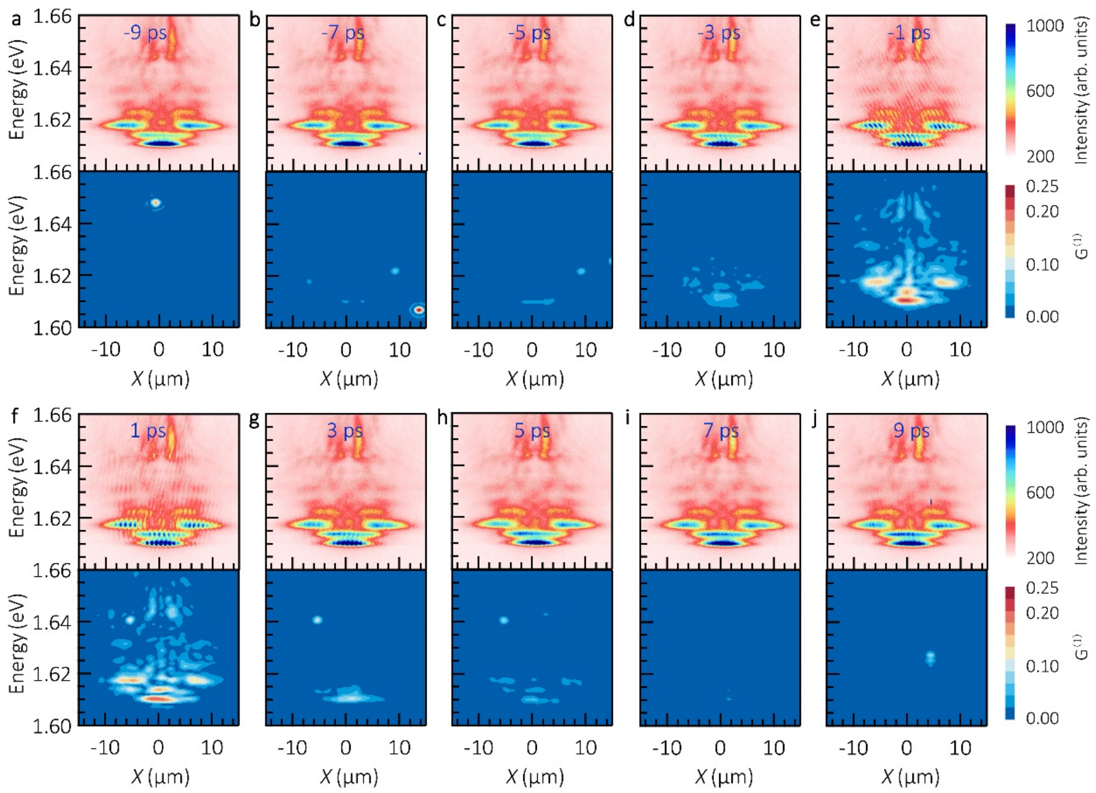

Supplementary Figure 7. First order autocorrelation function $G^{(1)}$ as a function of delay time. a-j Interference image (upper) and corresponding $G^{(1)}$ (down) of polaritons at different delay time, changing from -9 ps to 9 ps. The pump power is 90 μW.

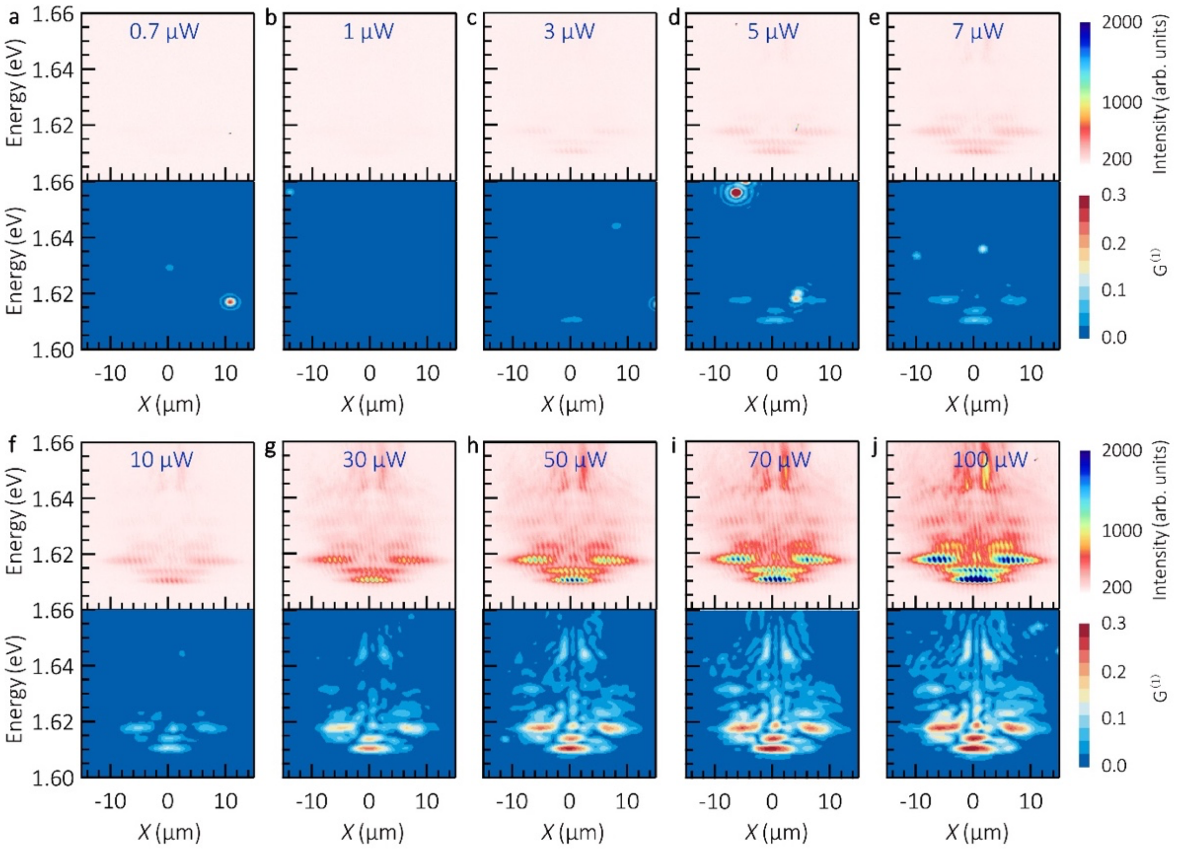

Supplementary Figure 8. First order autocorrelation function $G^{(1)}$ as a function of pump power. **a-j** Interference image (upper) and corresponding $G^{(1)}$ (down) of polaritons under different pump powers, changing from 0.7 μW to 100 μW. The delay time is set to zero. It can be found that a tiny impurity at interference image can result in an abnormal data point of $G^{(1)}$. The random impurity is induced by cosmic rays, recorded by the CCD during exposure time.

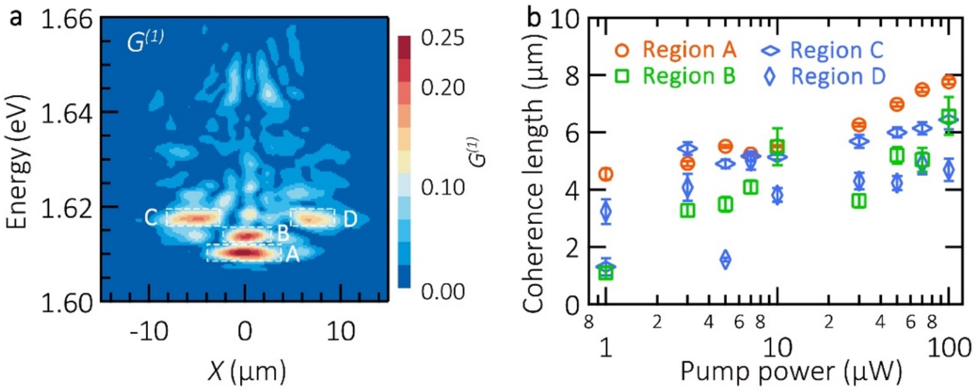

Supplementary Figure 9. Analysis of the coherence length in different trapped states. a $G^{(1)}$ map versus energy and position, this panel is reproduced from Fig. 4d of the main text. We label four regions A-D from the different polariton trap areas where the $G^{(1)}$ values are intense. **b** Corresponding coherence length from the A-D regions. It is worth to note that regions C and D are part of the same polariton trapped state. The error bars correspond to the 95% confidence interval of the Gaussian fitting from the $G^{(1)}$(x) spatial shape of the polariton trap.

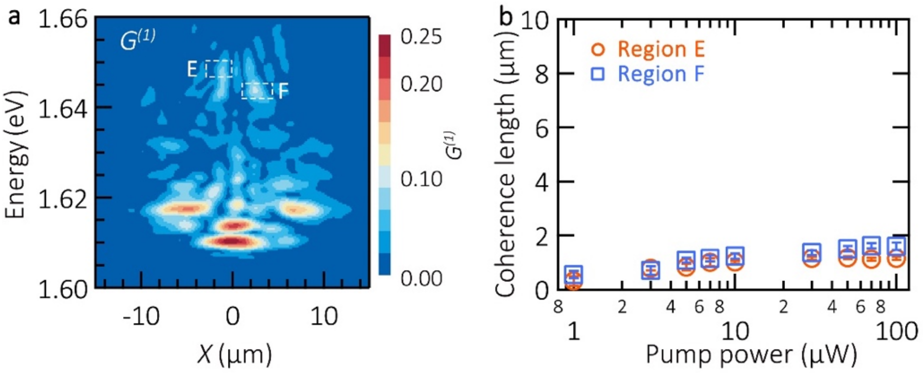

Supplementary Figure 10. Analysis of the coherence length of high energy states. **a** First-order correlation function map versus energy and real space, panel reproduced from Fig. 4d. The analysis regions are labelled as E and F. **b** Coherence lengths of regions E and F, saturating at a maximum of ~1.5 μm. The longest coherence length of the high energy state is shorter than the laser spot (~3 μm), and is comparable to the De Broglie wavelength of our room temperature polariton system (~1.2 μm). This suggests the absence of long spatial coherence of high energy states. The error bars correspond to the 95% confidence interval of the Gaussian fitting from the $G^{(1)}(x)$ spatial shape of the polariton trap.

S7. Quality factor of the empty cavity mode: reflectivity measures

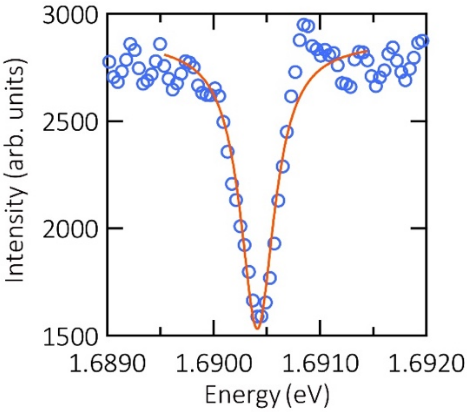

Supplementary Figure 11. Basic optical characterization of microcavity. Reflection spectrum of the microcavity at $K_{//}$=0 away from the WSe_2 monolayer. The resonance peak is at 1.6904 eV with FWHM of ~0.392 meV. The quality factor Q = 4300±400.

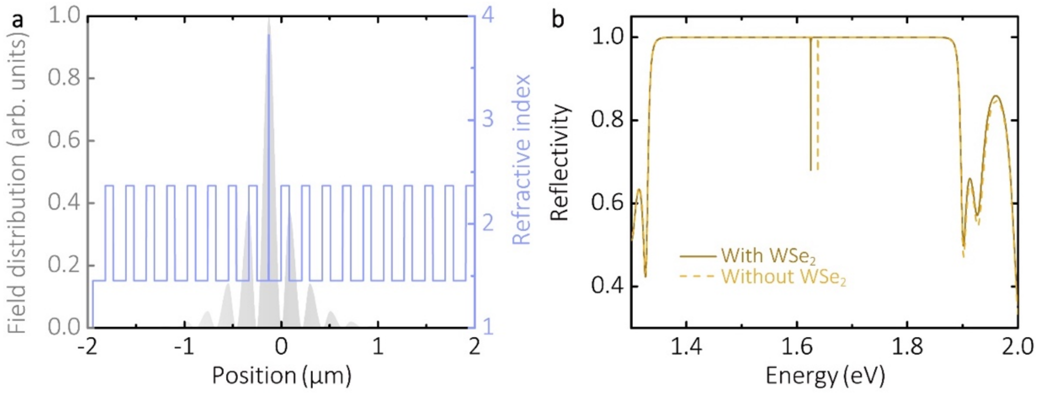

Supplementary Figure 12. Transfer matrix simulations on the microcavity structure: determination of the cavity mode. a Refractive index profile of the structure (with WSe_2), including the intensity of the confined cavity field at the resonance cavity energy 1.612 eV (normal emission from the cavity). **b** Simulated reflectivity versus energy without WSe_2 (dashed trace) and with WSe_2 (solid trace) considering no absorption. The refractive index of WSe_2 is extracted from Ref [9]. The refractive indexes of h-BN, SiO_2, TiO_2 are 1.61, 1.45 and 2.36, respectively. The spectral resolution in this simulation is set to describe accurately the reflectivity dip.

S8. Supplementary data on the magnetic response of room temperature polaritons

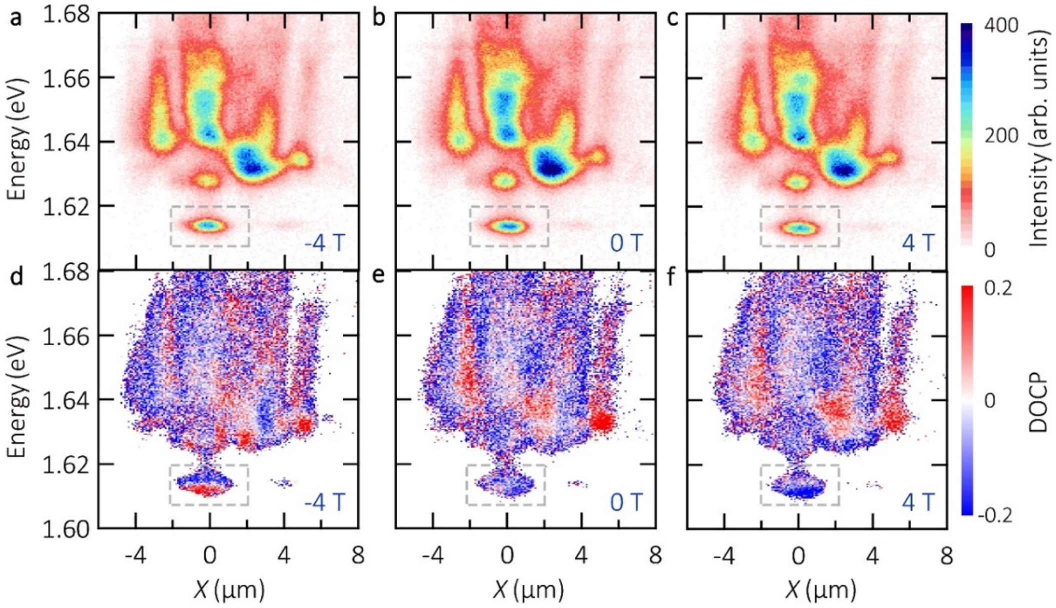

Supplementary Figure 13. PL distribution and circular polarisation at different magnetic fields. a-c Representative PL distribution ($I\sigma^{+}+I\sigma^{-}$) of polariton as a function of energy and real space at magnetic field of -4 T, 0 T and 4T, respectively. The pump power is 100 µW. **d-f** Corresponding DOCP images at magnetic field of -4 T, 0 T and 4T, respectively. For the sake of clarity in the data representation, we set a zero DOCP when the intensity of the dispersion relation is less than 10% of the maximum.

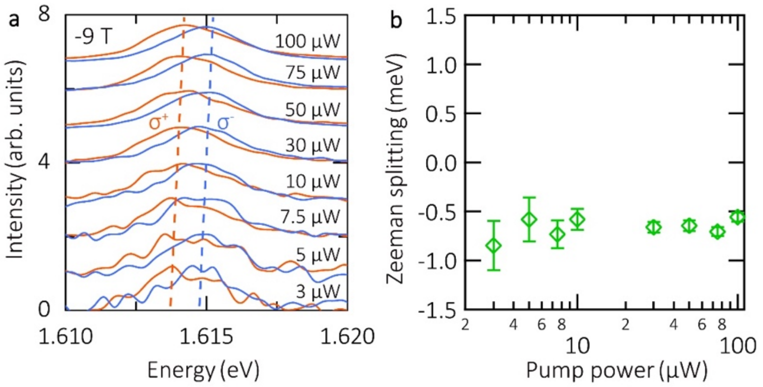

Supplementary Figure 14. Circular polarisation of room temperature polaritons as a of function of pump power at a magnetic field of -9 T. a Normalized PL intensity of σ^+ (red) and σ^- (blue) detection emission at a magnetic field of -9 T. **b** Corresponding Zeeman splitting under different excitation pump powers. The error bars correspond to the 95% confidence interval of the Lorentzian fit to the trapped polariton spectrum.

Supplementary references

1. Kaitouni, R. I. *et al.* Engineering the spatial confinement of exciton polaritons in semiconductors. *Phys. Rev. B* **74**, 155311 (2006).
2. Lundt, N. *et al.* Magnetic-field-induced splitting and polarization of monolayer-based valley exciton polaritons. *Phys. Rev. B* **100**, 121303 (2019).
3. Lundt, N. *et al.* Room-temperature Tamm-plasmon exciton-polaritons with a WSe2 monolayer. *Nat Commun* **7**, 13328 (2016).
4. Kavokin, A. V., Baumberg, J. J., Malpuech, G. & Laussy, F. P. *Microcavities*. vol. 1 (Oxford University Press, 2017).
5. Król, M. *et al.* Giant spin Meissner effect in a nonequilibrium exciton-polariton gas. *Phys. Rev. B* **99**, 115318 (2019).
6. Anton-Solanas, C. *et al.* Bosonic condensation of exciton–polaritons in an atomically thin crystal. *Nat. Mater.* **20**, 1233–1239 (2021).
7. Zhao, J. *et al.* Ultralow Threshold Polariton Condensate in a Monolayer Semiconductor Microcavity at Room Temperature. *Nano Lett.* **21**, 3331–3339 (2021).
8. Krizhanovskii, D. N. *et al.* Coexisting nonequilibrium condensates with long-range spatial coherence in semiconductor microcavities. *Phys. Rev. B* **80**, 045317 (2009).
9. Li, Y. *et al.* Measurement of the optical dielectric function of monolayer transition-metal dichalcogenides: MoS 2 , Mo S e 2 , WS 2 , and WS e 2. *Phys. Rev. B* **90**, 205422 (2014).
